# Generalized Index Coding Problem and Discrete Polymatroids †

**DOI:** 10.3390/e22060646

**Published:** 2020-06-10

**Authors:** Anoop Thomas, Balaji Sundar Rajan

**Affiliations:** 1 School of Electrical Sciences, Indian Institute of Technology Bhubaneswar, Odisha 752050, India; anoopthomas@iitbbs.ac.in; 2 Department of Electrical Communication Engineering, Indian Institute of Science Bangalore, Bangalore 560012, India

**Keywords:** index coding, generalized index coding problems, representable discrete polymatroids, matroids

## Abstract

The connections between index coding and matroid theory have been well studied in the recent past. Index coding solutions were first connected to multi linear representation of matroids. For vector linear index codes, discrete polymatroids, which can be viewed as a generalization of the matroids, were used. The index coding problem has been generalized recently to accommodate receivers that demand functions of messages and possess functions of messages. In this work we explore the connections between generalized index coding and discrete polymatroids. The conditions that need to be satisfied by a representable discrete polymatroid for a generalized index coding problem to have a vector linear solution is established. From a discrete polymatroid, an index coding problem with coded side information is constructed and it is shown that if the index coding problem has a certain optimal length solution then the discrete polymatroid is representable. If the generalized index coding problem is constructed from a matroid, it is shown that the index coding problem has a binary scalar linear solution of optimal length if and only if the matroid is binary representable.

## 1. Introduction

The broadcast nature of the wireless medium is utilized by many applications such as multimedia content delivery, audio and video on-demand and ad-hoc wireless networking. The index coding problem introduced by Birk and Kol [1] aims to increase the throughput of wireless networks. The model considered in [1] involves a source that possesses a set of messages and a set of receivers that demand messages. Each receiver knows a proper subset of messages, which is referred to as the side information. The source also knows the side information available to the receivers. It uses this knowledge to develop proper encoding techniques to satisfy the demands of the receivers at an increased throughput. The source needs to transmit functions of messages to ensure that the receivers are able to decode their demanded messages. An index code is an encoding scheme developed by the source to satisfy all the receivers. An encoding scheme with a minimum number of transmissions that enables all the receivers to decode its demanded messages is referred to as an optimal index code.

Bar-Yossef et al. [2] studied a special case of index coding problem and found that the length of the optimal linear index code is equal to the minrank of a related graph. Graph theory techniques were used to find optimal index codes for a certain class of index coding problems in [3,4]. The case in which the side information can be represented by a special structure, referred to as interlinked cycles, was studied in [5], and optimal codes were constructed for them in [6].

An instance of the conventional index coding problem involves a source that possesses all the messages and a set of receivers. Each receiver possesses a subset of messages called the side information or the *Has-set* and demands another subset of messages called the *Want-set*. The wireless broadcast channel is assumed to be noiseless. The source is aware of the messages possessed by each receiver and it aims to reduce the number of transmissions required to satisfy the demands of all the receivers.

The problem of index coding has been extended in many directions. The problem of index coding with the restricted information problem is introduced in [7]. In the index coding with the restricted information problem, for each receiver, there is a certain subset of messages that the receiver should not be able to decode, referred to as restricted messages. The source has to design encoding schemes that satisfy the demands of all the receivers while ensuring that no receiver will be able to decode its restricted messages. The problem of index coding with erroneous transmissions was studied by Dau et al. [8]. The problem of finding the minimum length index code that enables all the receivers to correct a specific number of errors is addressed. Error-correction is achieved by using extra transmissions. In [8], only the transmitted symbols were error prone. This was extended in [9], where the side-information possessed by the receiver is also error prone. In this paper, we consider another extension to the index coding problem referred to as the Generalized Index Coding (GIC) problem. The generalized index coding problem is a special case of the functional index coding problem introduced in [10]. In functional index coding, the receivers and the source possess functions of messages rather than the actual messages. The generalized index coding problem is a special case of functional index coding when the functions are restricted to be linear [11].

In a functional index coding problem, the *Has-set* and the *Want-set* may contain functions of messages rather than subsets of messages. Note that the conventional index coding problem is a special case of the functional index coding problem. The problem with the *Has-sets* being linear combinations of messages was studied in [11,12] where it was called index coding with coded side information. This was motivated by the fact that certain clients may fail to receive some coded transmissions possibly due to a power outage. The clients will now possess a few coded transmissions as side information and the new problem is an index coding problem with coded side information. Dai et al. [13] considered both *Has-sets* and *Want-sets* to be linear combinations of the messages and the corresponding index coding problem is referred to as the generalized index coding problem. For the generalized index coding problem, a generalized minrank parameter, which gives the optimal length for linear encodings, was found in [14]. The paper also considers error correcting index codes for generalized index coding problems and obtain bounds on the lengths of optimal index codes. For some specific class of generalized index coding problems, optimal error correcting index codes are found in [15].

Consider the scenario with one source and four receivers $R_{1},R_{2},R_{3}$ and $R_{4}$. The source node possesses four packets $X_{1},X_{2},X_{3}$ and $X_{4}$. Receiver $R_{i}$ wants packet $X_{i}$ for i=1,…,4. The packets in the *Has-set* of the receivers is as follows: $R_{1}$ has packet $X_{2}$, $R_{2}$ has packet $X_{1}$, $R_{3}$ has packet $X_{4}$ and $R_{4}$ has packet $X_{3}$. From the classical index coding problem, the source has to transmit two coded packets $X_{1}+X_{2}$ and $X_{3}+X_{4}$ to satisfy all receivers. The transmissions are over an erasure broadcast channel. Receivers $R_{1}$ and $R_{2}$ fail to receive $X_{1}+X_{2}$ but receive $X_{3}+X_{4}$. Similarly, receivers $R_{3}$ and $R_{4}$ fails to receive $X_{3}+X_{4}$ but receives $X_{1}+X_{2}$. At this point, if only classical index coding is considered, the source needs to transmit the two coded packets again. However, if we consider the coded packets available to the receivers then the source only needs to transmit $X_{1}+X_{2}+X_{3}+X_{4}$ to satisfy all the receivers. Hence, by using generalized index coding, the source is able to save one transmission.

Index coding also finds application in the field of coded caching [16]. Once the caching scheme is fixed, for a specific demand, the design of the delivery scheme becomes an index coding problem. If the contents of the cache are encoded, then any delivery scheme corresponds to a solution of the corresponding generalized index coding problem. The relationship between index coding and coded caching is made use of in [17,18] to show the optimality of uncoded caching schemes. In [19], index coding techniques are used for caching problems to obtain solutions meeting outer bounds. The connections between index coding and coded caching are also used to design optimal error correcting delivery schemes for coded caching problems in [20,21,22,23].

The equivalence between index coding problems and network coding problem is established in [24]. From a network coding problem, an index coding problem is constructed and it is shown that a linear solution to the network coding problem exists if and only if a linear solution to the index coding problem exists. These results were extended to the non-linear case in [25]. The connections between network coding and matroid theory is established in [26]. Using these connections, it was shown in [27] that non-linear solutions perform better than linear solutions for the general network coding problem. The connection between index coding problems and multi-linear representation of matroids was studied in [24]. It was shown in [28] that a vector linear solution to an index coding problem exists if and only if there exists a representable discrete polymatroid satisfying certain conditions, which are determined by the index coding problem. The relationship between the network computing problem and functional index coding problem is established in [29]. In this paper, we establish the connections between generalized index coding problems, which is a special class of functional index coding problems and discrete polymatroids. The major contributions of this paper are as follows.

- We establish a connection between vector linear index codes for a generalized index coding problem and representable discrete polymatroids. It is shown that the existence of a linear solution for a generalized index coding problem is connected to the existence of a representable discrete polymatroid satisfying certain conditions determined by the generalized index coding problem.
- From a discrete polymatroid, we construct a generalized index coding problem and show that if the generalized index coding problem has a vector linear solution of optimal length over the binary field then the discrete polymatroid is representable over the binary field. An example to illustrate that the converse of the above result is not true, is also provided.
- A generalized index coding problem is constructed from matroids and it is shown that the constructed problem has an optimal binary scalar linear solution if and only if the matroid is binary representable. This enables us to construct a generalized index coding problem from a binary representable matroid and the constructed index coding problem has an optimal binary scalar linear solution. Also, it is shown that certain generalized index coding problems do not have a binary scalar linear solution of optimal length using the above result.

**Figure 1:**
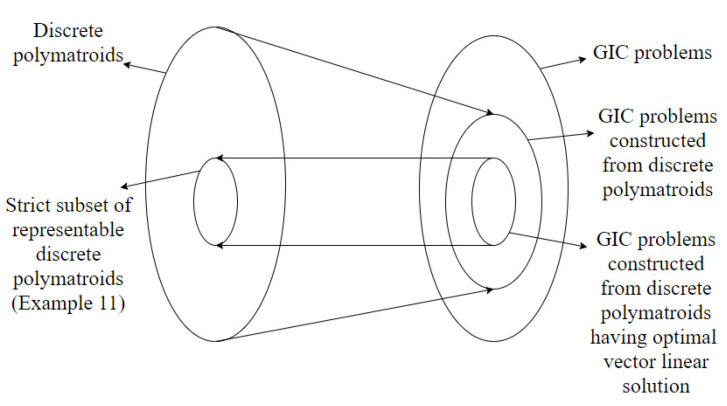
Diagram illustrating the connections between discrete polymatroids and generalized index coding problems.

**Figure 2:**
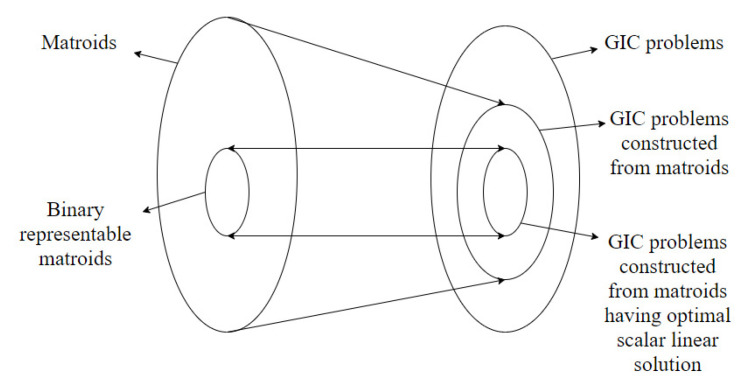
Diagram illustrating the connections between matroids and generalized index coding problems.

A part of the content of this paper was presented in [30]. In this journal version, the proofs of all the claims are added along with detailed examples. The results in this paper are an extension of the results in [24,28]. Specifically, Theorem 3 in [28] establishes the connections between a vector linear index code for an index coding problem and a representable discrete polymatroid. It is shown that a vector linear index coding solution exists if and only if there exists a discrete polymatroid satisfying certain conditions derived from the index coding problem. In this paper, we extend these results to the case of generalized index coding problems. The extension was established by modifying the conditions that the corresponding discrete polymatroid has to satisfy. We also note that the result in [28] can be obtained as a special case of the theorem derived in this paper. Starting from a discrete polymatroid, an index coding problem is constructed in Theorem 4 in [28]. The constructed index coding problem is shown to have an optimal perfect linear solution. In this paper, the construction is modified to get generalized index coding problems from discrete polymatroids. The paper identifies the modifications required from the existing scenario to get new results and also discusses the limitations of the extension by providing counter examples. In the case of conventional index coding problems, necessary and sufficient conditions for the constructed index coding problem to have an optimal linear solution is provided in terms of the representability of discrete polymatroids. For the generalized index coding solution, we show that representability is not guaranteed to provide optimal linear index coding solution via a counter example. Figure 1 summarizes the above results connecting discrete polymatroids and generalized index coding problems constructed from discrete polymatroids. The paper also identifies the modifications required to obtain a stronger result. This is achieved by restricting the construction to matroids rather than discrete polymatroids. This construction is similar to the construction in [24]. Specifically, Theorem 12 in [24] shows that the index coding problem constructed from a matroid has an optimal perfect linear solution if the matroid has an *n*-linear representation. In this paper, we show that the generalized index coding problem constructed from binary representable matroids has optimal perfect linear solutions. It is also shown the if the generalized index coding problem has an optimal perfect linear solution, the matroid is binary representable. The above result can be used in the construction of generalized index coding problems having optimal scalar linear solutions. Figure 2 summarizes the results connecting matroids and generalized index coding problems.

The organization of the paper is as follows. In Section 2, we present a short review on functional index coding. In Section 3, basic results of matroids and discrete polymatroids are reviewed. In Section 4, the connections between generalized index coding and discrete polymatroids are established. In Section 5, a generalized index coding problem is constructed from discrete polymatroids and it is shown that the index coding problem constructed has an optimal vector linear solution only if the discrete polymatroid is representable. In Section 6, similar construction is employed on matroids and shown that the constructed generalized index coding problem has an optimal binary scalar linear solution if and only if the matroid is binary representable. We conclude with a summary of results in Section 7 along with few directions for further research.

*Notations:* The set {1,2,…,m} is denoted as ⌈m⌋ and $Z_{\geq 0}$ denote the set of non-negative integers. A vector of length *r* in which the $i^{\mathrm{th}}$ component is one and all other components are zeros, denoted as $\epsilon_{i,r}$. For a vector *v* of length *r* and A⊆⌈r⌋,v(A) is the vector obtained by taking only the components of *v* indexed by the elements of *A*. For $u,v\in Z_{\geq 0}^{r},u\leq v$ if all the components of v−u are non-negative and u<v if u≤v and u≠v. For a set *S*, |S| denotes the cardinality of the set *S* and for a vector $v\in Z_{\geq 0}^{r},\left|v\right|$ denotes the absolute sum of components of *v*. For $u,v\in Z_{\geq 0}^{r},u\vee v$ is the vector in which the $i^{\mathrm{th}}$ component is the maximum of the $i^{\mathrm{th}}$ components of *u* and *v*. For a vector $v\in Z_{\geq 0}^{r},{\left(v\right)}_{>0}$ denotes the set of indices corresponding to the non-zero components of *v*. For a matrix *M*, $M_{i}$ denotes the $i^{\mathrm{th}}$ column of matrix *M* and for a set *S*, $M_{S}$ denotes the submatrix obtained by concatenating the columns of *M* indexed by the set *S*. For vector subspaces $V_{1},V_{2},\ldots ,V_{m}$ of a vector space *V*, the sum of vector spaces is the vector space $\sum_{i=1}^{m}V_{i}=\left\{\sum_{i=1}^{m}v_{i}\;|\;v_{i}\in V_{i}\right\}.$

## 2. Functional Index Coding

An index coding problem I(X,R) includes

- a set of messages $X=\{x_{1},x_{2},\ldots ,x_{m}\}$ and
- a set of receiver nodes R⊆{(x,H);x∈X,H⊆X∖{x}}.

For a receiver node R=(x,H)∈R, *x* denotes the message demanded by the receiver *R* and *H* denotes the side information possessed by *R*. Each one of the messages $x_{i},i\in \left\lceil m\right\rfloor$ belongs to $F_{q}$ where $F_{q}$ is the finite field with *q* elements. In an index coding problem, the source can take *n* instances of each message and encode them together such that each receiver is able to decode all *n* instances of the demanded messages.

An index code over $F_{q}$ of length *l* and dimension *n* for the index coding problem I(X,R) is a function $f:F_{q}^{mn}\to F_{q}^{l}$, which satisfies the following condition. For every receiver R=(x,H)∈R, there exists a function $\psi_{R}:F_{q}^{n|H|+l}\to F_{q}^{n}$ such that $\psi_{R}\left({\left(x_{i}\right)}_{i\in H},f\left(y\right)\right)=x,\forall y\in F_{q}^{mn}$. The function $\psi_{R}$ is referred to as the decoding function at the receiver *R*. An index coding solution for which n=1 is called scalar index code and if n>1, it is called a vector index code. An index code is called linear if the function *f* is linear.

The index coding problem was generalized to the functional index coding problem in [10]. In the functional index coding problem, the side information and the demands of the receivers may be functions of messages rather than only a subset of messages. The side information possessed by the receivers is described by a *Has-set*, which consists of functions of messages. The demands of the receiver are described by a *Want-set*. Each receiver $R_{i}$ is described by a tuple $\left(W_{i},H_{i}\right)$, where $W_{i},H_{i}$ are sets of functions from $F_{q}^{m}$ to $F_{q}$.

In this paper, we consider those generalized index coding problems for which the functions demanded and possessed by the receivers are linear combinations of the messages.

**Definition** **1.** *An instance I(X,R) of a generalized index coding problem comprises of*

*1.*  *A source equipped with the message vector $X=(x_{1},x_{2},\ldots ,x_{m})$, where $x_{i}\in F_{q},\forall \;i\in \left\lceil m\right\rfloor$.*
*2.*  *A set of clients or receivers $R=\{R_{1},R_{2},\ldots ,R_{\left|R\right|}\}$, where $R_{i}=\left(W_{i},H_{i}\right)$ for all $R_{i}\in R$. For any receiver $R_{i},H_{i}=\left\{h_{i,1}\left(X\right),h_{i,2}\left(X\right),\ldots ,h_{i,|H_{i}|}\left(X\right)\right\}$ is the Has-set, where $h_{i,j}:F_{q}^{m}\to F_{q}$ for $1\leq j\leq |H_{i}|$ and $W_{i}=\left\{w_{i,1}\left(X\right),w_{i,2}\left(X\right),\ldots ,w_{i,|W_{i}|}\left(X\right)\right\}$ is the Want-set, where $w_{i,k}:F_{q}^{m}\to F_{q}$ for $1\leq k\leq |W_{i}|$.*

The source can combine *n* instances of the messages and perform encoding operations such that the demands of the receivers are satisfied. Since the functions in the *Has-set* of a receiver $R_{i}$ are linear it can be represented as an inner product as follows. Each function $h_{i,j}\in H_{i}$ can be expressed as the inner product $h_{i,j}\left(X\right)=XK_{i,j}$ where $K_{i,j}\in F_{q}^{mn\times n}$ is a matrix. For the receiver $R_{i}$, we have $|H_{i}|$ functions in the *Has-set*, each represented by a matrix $K_{i,j},1\leq j\leq \left|H_{i}\right|$. All the functions in the *Has-set* of receiver $R_{i}$ can be represented by a matrix $K_{i}\in F_{q}^{mn\times n|H_{i}|}$ called the *knowledge matrix*. Note that $K_{i}=\left[K_{i,1},K_{i,2},\ldots ,K_{i,|H_{i}|}\right]$. Similarly, the demand functions in the *Want-set* $W_{i}$ can be represented by *demand matrices*. Each function $w_{i,j}\in W_{i}$ can be expressed as $w_{i,j}\left(X\right)=XD_{i,j}$ where the matrix $D_{i,j}\in F_{q}^{mn\times n}$ and all the functions in the *Want-set* of receiver $R_{i}$ can be described by the $mn\times n|W_{i}|$ matrix $D_{i}=\left[D_{i,1},D_{i,2},\ldots ,D_{i,|W_{i}|}\right]$ called the *demand matrix*.

An index code over $F_{q}$ of length *l* and dimension *n* for the generalized index coding problem I(X,R) is a function $f:F_{q}^{mn}\to F_{q}^{l}$, which satisfies the following condition. For every receiver $R_{i}=\left(W_{i},H_{i}\right)\in R$, there exists a function $\psi_{R_{i}}:F_{q}^{n|H_{i}|+l}\to F_{q}^{n|W_{i}|}$ such that $\psi_{R_{i}}\left(XK_{i},f\left(X\right)\right)=XD_{i},\forall X\in F_{q}^{mn}$. The definitions of linearity, scalar and vector index codes remains the same as that of conventional index codes.

When the index code *f* for a generalized index coding problem is linear it can be described as $f\left(X\right)=XL,\forall X\in F_{q}^{mn}$, where *L* is a matrix of order mn×l over $F_{q}$. The matrix *L* is called as the matrix corresponding to the linear index code *f* and the code *f* is referred to as the linear index code based on *L*.

For an index coding problem I(X,R), define μ(I(X,R)) as the maximum number of receivers having the same *Has-set*. The length *l* and dimension *n* of an index coding solution for the index coding problem I(X,R) satisfy the condition l/n≥μ(I(X,R)) [24]. Computing the optimal length of an index coding solution is shown to be an NP-hard problem [31]. The lower bound offers a method to check whether the solution obtained is optimal.

**Definition** **2** ([24])**.** *An index coding solution for which l/n=μ(I(X,R)) is defined to be a perfect index coding solution.*

**Example** **1.** *Consider the generalized index coding problem with the message vector $X=[x_{1}\;x_{2}\;\ldots \;x_{5}],$$x_{i}\in F_{2}.$ There are five receivers $R_{1}=\left(x_{1},\left\{x_{2}\right\}\right),R_{2}=\left(x_{2},\left\{x_{1}+x_{5}\right\}\right),R_{3}=\left(x_{3},\left\{x_{1},x_{4}\right\}\right),R_{4}=\left(x_{4},\left\{x_{1}+x_{2}+x_{3}\right\}\right)$ and $R_{5}=\left(x_{5}+x_{4}+x_{3},\left\{x_{2},x_{1}+x_{3}\right\}\right)$. Consider receiver $R_{5}=\left(W_{5},H_{5}\right).$ The knowledge matrix $K_{5}$ and the demand matrix $D_{5}$ are as follows.*

$$K_{5}=\left[\begin{array}{cc} 0 & 1\\ 1 & 0\\ 0 & 1\\ 0 & 0\\ 0 & 0 \end{array}\right],D_{5}=\left[\begin{array}{c} 0\\ 0\\ 1\\ 1\\ 1 \end{array}\right].$$

*The source can satisfy the demands of all the receivers by transmitting three messages $x_{1}+x_{2},x_{3}+x_{4}$ and $x_{5}$. The index code is scalar linear and is described by the matrix*

$$L=\left[\begin{array}{ccc} 1 & 0 & 0\\ 1 & 0 & 0\\ 0 & 1 & 0\\ 0 & 1 & 0\\ 0 & 0 & 1 \end{array}\right].$$

## 3. Matroids and Discrete Polymatroids

Matroids are mathematical structures that capture the fundamental properties of dependence, which are common to graphs and matrices. In [24], the connections between network coding, index coding and multi-linear representation of matroids are established. It was observed that each receiver imposes a certain dependency between the index coding solutions, side information and the demand. This dependency arises from the fact that, using the transmitted messages of an index coding solution, along with the messages present as side information, each receiver should be able to decode the demanded messages. It was observed in [28] that matroids cannot fully capture all the dependencies of a vector linear index coding solution, and discrete polymatroids having a more general structure were used to establish these connections. In this paper, we use discrete polymatroids to capture the dependencies of the generalized index coding problem and also show that the problem of finding a representation for a discrete polymatroid can be reduced to the problem of obtaining an optimal perfect linear index coding solution. In Section 3.1 and Section 3.2, we review the definitions and establish notations related to matroids and discrete polymatroids. In Section 3.2, we review how discrete polymatroids can be viewed as a generalization of matroids.

### 3.1. Matroids

In this subsection, we list a few basic definitions and results from the matroid theory. For a comprehensive treatment, the readers are referred to [32,33].

**Definition** **3.** *Let E be a finite set. A matroid M on E is an ordered pair (E,I), where the set I is a collection of subsets of E satisfying the following three conditions*

*(I1)*  ϕ∈I
*(I2)*  *If X∈I and $X^{\prime }\subseteq X$, then $X^{\prime }\in I$.*
*(I3)*  *If $X_{1}$ and $X_{2}$ are in I and $|X_{1}\left|<\right|X_{2}|$, then there is an element $e\in X_{2}-X_{1}$ such that $X_{1}\cup e\in I$.*

The set *E* is called the *ground set* of the matroid and is also referred to as E(M). The members of set I are called the *independent sets* of M. Independent sets are also denoted by I(M). The set of independent sets in a matroid generalizes the notion of linear independence in vectors of a vector space. Note that the properties (I2) when specialized to the vectors of a vector space implies that a subset of a linearly independent set is linearly independent. Property (I3) is a generalization of the extension of a linearly independent set by using vectors from a larger linearly independent set. Similar to a vector space, a matroid can also be defined in different ways. The notion of the basis of a vector space and the dependent vectors of a vector space is also generalized in matroids and is provided below.

A maximal independent subset of *E* is called a *basis* of M and the set of all bases of M is denoted by B(M). A subset of *E* that is not in I is called a *dependent set*. A minimal dependent set C⊆E is referred to as a *circuit*. The set of all circuits of matroid M is denoted by C(M). The circuits of a matroid satisfy the following conditions.

(C1) No proper subset of a circuit is a circuit.
(C2) If $C_{1}$ and $C_{2}$ are distinct circuits and $c\in C_{1}\cap C_{2}$, then $C_{1}\cup C_{2}\setminus \left\{c\right\}$ contains a circuit

The axioms (C1) and (C2) can be viewed as generalizations of the properties of a minimal dependent set in a vector space. The axiom (C1) implies that any subset of a circuit is independent and is easily proved using the minimality given in the definition. The axiom (C2) shows that if there are two minimally dependent sets with a vector in common, then the union of two sets removing the common vector is a dependent set. This follows from the fact that the common vector can be expressed as combinations of other vectors in $C_{1}$ and $C_{2}$, respectively, and thus establishing a dependency between the vectors in $C_{1}\cup C_{2}\setminus \left\{c\right\}$. The notion of rank in a vector space is also generalized in the matroid as given below.

Each circuit of a matroid is a set that captures the dependencies existing in a matroid. An index coding problem induces certain dependencies. However, these dependencies are not minimal. Rather than trying to find the minimal dependent sets, a common approach is to use the notion of a rank function of matroid defined below to characterize the dependencies.

A function called the *rank* function is associated, the domain of which is the power set of *E* and codomain is the set of non-negative integers. The rank of any X⊆E in M, denoted by $r_{M}\left(X\right)$ is defined as the maximum cardinality of a subset of *X* that is a member of I(M). The rank of matroid is the rank of its ground set. The rank function of the matroid satisfies the following properties.

(R1) $r_{M}\left(X\right)\leq \left|X\right|$, for all X⊆E.
(R2) $r_{M}\left(X\right)\leq r_{M}\left(Y\right)$, for all X⊆Y⊆E.
(R3) $r_{M}\left(X\cup Y\right)+r_{M}\left(X\cap Y\right)\leq r_{M}\left(X\right)+r_{M}\left(Y\right)$, for all X,Y⊆E.

Note that the rank of an independent set is equal to the cardinality of the independent set. A matroid is fully described by its rank function and a matroid M on ground set *E* with rank function $r_{M}$ denoted as $M(E,r_{M})$.

**Example** **2.** *Consider the matroid M on the ground set ⌈4⌋ with the rank function $r_{M}$ defined as $r_{M}\left(X\right)=\min\left\{|X|,2\right\},X\subseteq \left\lceil 4\right\rfloor$. It follows from the definition of the rank function that the rank of an independent set is equal to the cardinality of the set. It also follows that any set with cardinality equal to the rank is an independent set. The set of independent sets of the matroid M is I(M)={ϕ,{1},{2},{3},{4},{1,2},{1,3},{1,4},{2,3},{2,4},{3,4}}. This matroid is referred to as the uniform matroi $U_{2,4}$. The rank of the matroid is $r_{M}\left(\left\lceil 4\right\rfloor \right)=2$. The set of circuits of the matroid is C(M)={X⊆⌈4⌋:|X|=3}. The set of all bases of M is B(M)={X⊆⌈4⌋:|X|=2}.*

A matroid M is said to be representable over $F_{q}$ if there exists one-dimensional vector subspaces $V_{1},V_{2},\cdots ,V_{\left|E\right|}$ of a vector space *V* such that $\dim\left(\sum_{i\in X}V_{i}\right)=r_{M}\left(X\right),\forall X\subseteq E$ and the set of vector subspaces $V_{i},i\in \left\lceil |E|\right\rfloor ,$ is said to form a representation of M. The one-dimensional vector subspaces $V_{i},i\in \left\lceil |E|\right\rfloor ,$ can be described by a matrix *A* over $F_{q}$, the $i^{\mathrm{th}}$ column of which spans $V_{i}.$ A matroid M with matrix *A* as its representation is called the vector matroid of *A* and is denoted by M(A). Each element in the ground set of M(A) corresponds to a column in *A*. For a subset *S* of the ground set E(M), $A_{S}$ denotes the submatrix of *A* with columns corresponding to the elements of the ground set in *S*.

**Example** **3.** *For the matroid considered in Example 2, consider the matrix $A=\left[\begin{array}{cccc} 1 & 0 & 1 & 1\\ 0 & 1 & 1 & 2 \end{array}\right]$. Let $V_{i},i\in \left\lceil 4\right\rfloor$ denote the space spanned by the $i^{th}$ column of A over $F_{3}$. It can be observed that any two columns of the matrix are linearly independent. Hence, for any set X⊂⌈4⌋, such that |X|≤2, dim$\left(\sum_{i\in X}V_{i}\right)=\left|X\right|$. Note that the number of rows of the matrix is 2 and hence dim$\left(\sum_{i\in X}V_{i}\right)=2,\forall X\subseteq \left\lceil 4\right\rfloor ,\left|X\right|\geq 3.$ Hence, the matrix A is a representation of the matroid.*

It was established in [24], that the problem of finding a multi-linear representation of matroids can be reduced to finding an optimal perfect linear index code for a corresponding index coding problem. Multi-linear representation of matroids was introduced in [34,35]. A matroid M on the ground set *E* is said to be multi-linearly representable of dimension *n* over $F_{q}$ if there exist vector subspaces $V_{1},V_{2},\ldots ,V_{\left|E\right|}$ of a vector space *V* over $F_{q}$ such that $\dim\left(\sum_{i\in X}V_{i}\right)=n\;r_{M}\left(X\right),\forall X\subseteq E$. The vector subspaces $V_{1},V_{2},\ldots ,V_{\left|E\right|}$ are said to form a multi-linear representation of dimension *n* over $F_{q}$ for the matroid M. The vector subspaces $V_{i},i\in \left\lceil |E|\right\rfloor$ can be described by matrices $M_{1},M_{2},\ldots ,M_{\left|E\right|}$ of order nk×k over $F_{q}$, where *k* is the rank of the matroid. Let *M* be the matrix obtained by concatenating the matrices $M_{1},M_{2},\ldots ,M_{\left|E\right|}$, $M=[M_{1}\;M_{2}\;\ldots \;M_{\left|E\right|}]$. For every subset X⊆E, rank $\left(M_{X}\right)=nr_{M}\left(X\right)$.

### 3.2. Discrete Polymatroids

Discrete polymatroids are multi-set analog to matroids. Linear representations of discrete polymatroids generalize the notion of linear and multi-linear representation of matroids. In this paper, we establish connections between vector linear index codes for the generalized index coding problem and representations of discrete polymatroids. In this subsection, we review the definitions and results from discrete polymatroids. For a comprehensive treatment, interested readers are referred to [36,37].

**Definition** **4** ([36])**.** *A discrete polymatroid D on the ground set ⌈m⌋ is a non-empty finite set of vectors in $Z_{\geq 0}^{m}$ satisfying the following conditions:*

- If u∈D and v<u, then v∈D.
- For all u,v∈D with |u|<|v|, there exists w∈D such that u<w≤u∨v.

Let $2^{\left\lceil m\right\rfloor }$ denote the power set of the set ⌈m⌋. For a discrete polymatroid D, the rank function $\rho :2^{\left\lceil m\right\rfloor }\to Z_{\geq 0}$ is defined as ρ(A)=max{|u(A)|,u∈D}, where ∅≠A⊆⌈m⌋ and ρ(∅)=0. Alternatively, a discrete polymatroid D can be written in terms of its rank function as $D=\{x\in Z_{\geq 0}^{m}:|x\left(A\right)|\leq \rho \left(A\right),\forall A\subseteq \left\lceil m\right\rfloor \}.$ A discrete polymatroid is completely described by the rank function. So the discrete polymatroid D on ⌈m⌋ is also denoted by (⌈m⌋,ρ). The ground set of discrete polymatroid is also denoted by E(D).

A function $\rho :2^{\left\lceil m\right\rfloor }\to Z_{\geq 0}$ is the rank function of a discrete polymatroid if and only if it satisfies the following conditions [38]:

(D1) For A⊆B⊆⌈m⌋,ρ(A)≤ρ(B).
(D2) ∀A,B⊆⌈m⌋,ρ(A∪B)+ρ(A∩B)≤ρ(A)+ρ(B).
(D3) ρ(∅)=0.

The difference between discrete polymatroids and matroids is better understood by comparing the properties of the rank functions of each of these structures. It can be observed that the main difference is that the rank of a matroid $r_{M}$ has to satisfy the additional property $r_{M}\left(X\right)\leq \left|X\right|,\forall X\subseteq E\left(M\right)$. This restriction is generalized and the advantage is that each element in the ground set can have different values for the rank. In particular, when the rank of every element in the ground set becomes one, the structure reduces to a matroid and when the rank of every element becomes n>1, it reduces to a matroid with multi-linear representation. This additional generalization is required to fully capture all the dependencies of a generalized index coding problem and this is illustrated in Theorem 1 in Section 4.

The notion of basis and circuits is also extended to the case of discrete polymatroids. A vector u∈D for which there does not exist v∈D such that u<v, is called a basis vector of D. Let B(D) denote the set of basis vectors of D. The sum of the components of a basis vector of D is referred to as the rank of D, denoted by ρ(D). Note that ρ(D)=ρ(⌈m⌋). For all the basis vectors, the sum of the components will be equal [37]. A discrete polymatroid can also be defined as the set of all integral subvectors of its basis vectors.

**Example** **4.** *Consider a discrete polymatroid on the ground set ⌈3⌋ defined by the set of basis vectors B(D)={(1,1,1),(1,2,0),(2,0,1),(2,1,0)}. The set of vectors belonging to the discrete polymatroid is the integral subvectors of its basis vectors. Hence, the discrete polymatroid D is {(0,0,0),(1,0,0),(0,1,0),(0,0,1),(1,0,1),(1,1,0),(0,1,1),(1,1,1),(2,0,0),(2,0,1),(2,1,0),(1,2,0),(0,2,0)}. The rank function ρ of the discrete polymatroid is given by ρ({1})=ρ({2})=ρ({2,3})=2, ρ({3})=1 and ρ({1,2})=ρ({1,3})=ρ({1,2,3})=3. It can be verified that the rank function satisfies the axioms (D1), (D2) and (D3).*

Consider a discrete polymatroid D with rank function ρ on the ground set ⌈m⌋. Consider the function $\rho^{\prime }\left(X\right)=n\rho \left(X\right),\forall X\subseteq \left\lceil m\right\rfloor$. The function $\rho^{\prime }$ satisfies the conditions (D1), (D2) and (D3). The discrete polymatroid on the ground set ⌈m⌋ with the rank function $\rho^{\prime }$ is denoted by nD.

**Definition** **5** ([38])**.** *A discrete polymatroid D on the ground set ⌈m⌋ with rank function ρ is said to be representable over $F_{q}$ if there exists vector subspaces $V_{1},V_{2},\cdots ,V_{m}$ of a vector space E over $F_{q}$ such that $dim\left(\sum_{i\in X}V_{i}\right)=\rho \left(X\right),$∀X⊆⌈m⌋. The set of vector subspaces $V_{i},i\in \left\lceil m\right\rfloor ,$ is said to form a representation of D. A discrete polymatroid is said to be representable if it is representable over some field. Each $V_{i}$ can be expressed as the column span of a ρ(⌈m⌋)×ρ({i}) matrix $A_{i}$. The concatenated matrix $A=[A_{1}\;A_{2}\ldots A_{m}]$ is referred to as the representing matrix of the discrete polymatroid D. It is shown in [39], that performing elementary row operations or column operations on A does not change the discrete polymatroid. In particular, pre multiplication and post multiplication by full rank matrices does not change the discrete polymatroid.*

*A discrete polymatroid can be constructed from any finite set of vector subspaces of a vector space. Let $V_{1},V_{2},\ldots ,V_{m}$ be a collection of vector subspaces of a vector space V. For any subset S⊆⌈m⌋, define $r\left(A\right)=dim\left(\sum_{i\in A}V_{i}\right)$. The function r satisfies the conditions (D1), (D2) and (D3) and hence it is the rank function of a discrete polymatroid on the ground set ⌈m⌋. Let $D(V_{1},V_{2},\ldots ,V_{m})$ denote the representable discrete polymatroid on ⌈m⌋ with $V_{1},V_{2},\ldots ,V_{m}$ as its representation.*

**Example** **5.** *Consider the set of matrices $A_{1}=\left[\begin{array}{cc} 1 & 0\\ 0 & 1\\ 0 & 0 \end{array}\right]$, $A_{2}=\left[\begin{array}{cc} 0 & 1\\ 0 & 1\\ 1 & 1 \end{array}\right]$ and $A_{3}=\left[\begin{array}{c} 0\\ 0\\ 1 \end{array}\right]$ over $F_{2}$. Let $V_{i},i\in \left\lceil 3\right\rfloor$ denote the column span of $A_{i}$. The set of vector spaces $V_{i},i\in \left\lceil 3\right\rfloor$ is a representation for the discrete polymatroid in Example 4.*

**Definition** **6** ([28])**.** *For a discrete polymatroid D with rank function ρ on the ground set ⌈m⌋, a vector $u\in Z_{\geq 0}^{m}$ is said to be an excluded vector if the $i^{\mathrm{th}}$ component of u is less than or equal to ρ({i}),∀i∈⌈m⌋ and u∉D. The set of excluded vectors for the discrete polymatroid D is denoted by D(D). An excluded vector u∈D(D) is said to be a minimal excluded vector, if there does not exist v∈D(D) for which v<u. The set of minimal excluded vectors for the discrete polymatroid D is denoted by C(D).*

Discrete polymatroids can be viewed as a generalization of matroids [28,36,37]. There is a one-to-one correspondence between the independent sets, basis sets, dependent sets and circuits of a matroid to the vectors of an associated discrete polymatroid. For a matroid M there is an associated discrete polymatroid D(M). This arises from the fact that the rank function of a matroid M satisfies the conditions (D1), (D2) and (D3). Hence, the rank function of matroid M also serves as the rank function of a corresponding discrete polymatroid D(M). Consider an independent set *I* of the matroid M. Corresponding to the set *I* there exists a unique vector $\sum_{i\in I}\epsilon_{i,r}$ belonging to D(M). Discrete polymatroid D(M) can be written as $\left\{\sum_{i\in I}\epsilon_{i,r}:I\in I\right\}$ where I is the set of independent sets of matroid M. For a basis set *B* of a matroid M, the vector $\sum_{i\in B}\epsilon_{i,r}$ is a basis vector of D(M) and for a basis vector *b* of D(M), the set ${\left(b\right)}_{>0}$ is a basis set of M. For a dependent set *D* of M, the vector $\sum_{i\in D}\epsilon_{i,r}$ is an excluded vector of D(M) and conversely for an excluded vector d∈D(D(M)), the set ${\left(d\right)}_{>0}$ is a dependent set of M. Similarly the set of minimal excluded vectors of D(M) and circuits of M are also related as follows. The set of circuits of matroid M is given by $\left\{{\left(u\right)}_{>0}:u\in C\left(D\left(M\right)\right)\right\}$. For a circuit *C* of matroid M the vector $\sum_{i\in C}\epsilon_{i,r}$ is a minimal excluded vector for D(M).

**Example** **6.** *Consider the uniform matroid $U_{2,4}$ considered in Example 2. The discrete polymatroid $D(U_{2,4})$ is*

$$\begin{array}{c} \{(0,0,0,0),(1,0,0,0),(0,1,0,0),(0,0,1,0),(0,0,0,1),(1,1,0,0),\\ (1,0,1,0),(1,0,0,1),(0,1,1,0),(0,1,0,1),(0,0,1,1)\}. \end{array}$$

*The set of vectors belonging to the discrete polymatroid $D(U_{2,4})$ can be obtained from the independent sets of the matroid. The set of excluded vectors can be obtained from the dependent sets. The set of excluded vectors for $D(U_{2,4})$ is*

$$\begin{array}{c} \{(1,1,1,0),(1,1,0,1),(1,0,1,1),(0,1,1,1),(1,1,1,1)\}. \end{array}$$

*The set of minimal excluded vectors of $D(U_{2,4})$ corresponds to the circuits of the matroid $U_{2,4}$. The set of minimal excluded vectors of $D(U_{2,4})$ is given by {(1,1,1,0),(1,1,0,1),(1,0,1,1),(0,1,1,1)}.*

## 4. Generalized Index Coding Problem and Discrete Polymatroids

In this section, we explore the connections between the generalized index coding problem and representable discrete polymatroids. Theorem 1 below connects the existence of a linear index code of length *l* and dimension *n* for a generalized index coding problem to the problem of representation of a discrete polymatroid satisfying certain conditions.

**Theorem** **1.** *A linear index code over $F_{q}$ of length l and dimension n exists for a generalized index coding problem I(X,R) if and only if there exists a discrete polymatroid D=(⌈m+1⌋,ρ) representable over $F_{q}$ with ρ(D)=mn and with $A_{1},A_{2},\ldots ,A_{m+1},$ as the representation matrices satisfying the following conditions: *

*(C1)*  *ρ({i})=n,∀i∈⌈m⌋,ρ(⌈m⌋)=mn and ρ({m+1})=l.*
*(C2)*  *For every receiver $R_{i}=\left(W_{i},H_{i}\right)\in R$ described by $\left(D_{i},K_{i}\right)$, rank $\left(\left[AD_{i}\;\;AK_{i}\;\;A_{m+1}\right]\right)=\;\mathrm{rank}\;\left(\left[AK_{i}\;\;A_{m+1}\right]\right)$, where $A=[A_{1}\;A_{2}\ldots A_{m}]$.*

**Proof.**  First we prove the if part. Consider a discrete polymatroid D of rank mn representable over $F_{q}$ with representation $A_{1},A_{2},\ldots ,A_{m+1},$ satisfying conditions (C1) and (C2). The matrix *A* is the concatenation of matrices $A_{1},A_{2},\ldots ,A_{m}$. Condition (C1) implies that $A_{i}$ is mn×n matrix for i∈⌈m⌋ and $A_{m+1}$ is mn×l matrix. From (C1) we have that rank(A)=mn making it invertible. Define $A_{i}^{\prime }=A^{-1}A_{i},i\in \left\lceil m+1\right\rfloor$. Consider the map $f:F_{q}^{mn}\to F_{q}^{l}$ given by $f\left(X\right)=XA_{m+1}^{\prime }$. We show that the map *f* forms an index code of length *l* and dimension *n* over $F_{q}$. Consider any receiver $R_{i}=\left(W_{i},H_{i}\right)$ described by $\left(D_{i},K_{i}\right)$. From (C2) we have that the column span of the matrix $AD_{i}$ belongs to the span of columns of $AK_{i}$ and $A_{m+1}$. Matrix $AD_{i}$ can be written as $\left[AK_{i}\;\;A_{m+1}\right]M_{i}$ where $M_{i}$ is an $\left(\right|H_{i}\left|+l)\times \right|W_{i}|$ matrix. Pre multiplying by $A^{-1}$, we have $\left[K_{i}\;\;A_{m+1}^{\prime }\right]M_{i}=D_{i}$. Hence $XD_{i}$ can be obtained at receiver $R_{i}$ from $XK_{i}$ and $XA_{m+1}^{\prime }$.

To prove the only if part, we assume that a vector linear index code *f* over $F_{q}$ of length *l* and dimension *n* exists for the generalized index coding problem I(X,R). The vector linear index code *f* can be written as $f\left(X\right)=XA_{m+1}$ where $A_{m+1}$ is a matrix of size mn×l. Let *I* be the identity matrix of size mn×mn. For i∈⌈m⌋, let $A_{i}$ be the matrix obtained by taking only the ${\left(i\left(n-1\right)+1\right)}^{th}$ to ${\left(in\right)}^{th}$ columns of *I*. Let $V_{i}$ be the column span of $A_{i}$. Consider the discrete polymatroid $D(V_{1},V_{2},\ldots ,V_{m+1})$. We claim that the discrete polymatroid $D(V_{1},V_{2},\ldots ,V_{m+1})$ satisfies the condition (C1) and (C2). Since the concatenation of matrices $A_{i},i\in \left\lceil m\right\rfloor$ forms an identity matrix, condition (C1) is satisfied. Consider a receiver $(D_{i},K_{i})\in R$. Since the vector index code $XA_{m+1}$ satisfies the receiver, $XD_{i}$ can be obtained from $XK_{i}$ and $XA_{m+1}$. Since *A* is the identity matrix, condition (C2) is satisfied. □

Theorem 1 is a generalization of the result obtained in [28] where the vector linear solution of a conventional index coding problem was connected to discrete polymatroids. The fact that the source and receiver possess functions of messages changes condition (2) in Theorem 1. The concept of the representing matrix is used to extend the result in [28]. The result in [28] can be obtained from this result by imposing the restriction on the structure of matrices $D_{i}$ and $K_{i}$.

**Corollary** **1.** *Corresponding to a receiver $R_{i}=\left(x_{i},H_{i}\right)$ of the conventional index coding problem, in which receiver $R_{i}$ demands the message $x_{i}$ and possess a subset of messages $H_{i}=\left\{x_{j_{1}},x_{j_{2}},\ldots ,x_{j_{k}}\right\}$ as its side information, condition (C2) reduces to $\rho \left(\left\{i\right\}\cup \left\{j_{1},j_{2},\ldots ,j_{k}\right\}\cup \left\{m+1\right\}\right)=\rho \left(\left\{j_{1},j_{2},\ldots ,j_{k}\right\}\cup \left\{m+1\right\}\right).$*

For conventional index coding, the matrix $D_{i}$ reduces to a vector with one non-zero entry corresponding to the demanded message. The matrix $K_{i}$ takes the structure of a submatrix of the identity matrix. Hence, $AD_{i}$ becomes $A_{i}$ and $AK_{i}$ corresponds to certain columns of the matrix *A*. The columns of the matrix *A* corresponds to the entries of the discrete polymatroid. By imposing the restrictions, condition (C2) can be expressed in terms of the elements of the ground set.

The necessary and sufficient conditions for a matrix *L* to correspond to a linear index code for a generalized index coding problem I(X,R) was found in [14]. Theorem 1 expresses the above condition in terms of properties of the corresponding discrete polymatroid D=(⌈m+1⌋,ρ). In the remaining part of this section, we illustrate Theorem 1 with examples.

**Example** **7.** *Consider the generalized index coding problem of Example 1. There are five messages and since the solution is scalar, dimension is one. Consider the set of matrices*

$$A_{1}=\left[\begin{array}{c} 1\\ 0\\ 0\\ 0\\ 0 \end{array}\right],A_{2}=\left[\begin{array}{c} 0\\ 1\\ 0\\ 0\\ 0 \end{array}\right],A_{3}=\left[\begin{array}{c} 0\\ 0\\ 1\\ 0\\ 0 \end{array}\right],A_{4}=\left[\begin{array}{c} 0\\ 0\\ 0\\ 1\\ 0 \end{array}\right],A_{5}=\left[\begin{array}{c} 0\\ 0\\ 0\\ 0\\ 1 \end{array}\right].$$

*Also let $A_{6}=L$, the matrix corresponding to the index code of Example 1. Let $V_{i}$ denote the column span of $A_{i}$ for i∈⌈6⌋. The discrete polymatroid $D(V_{1},V_{2},\ldots ,V_{6})$ satisfies the conditions (C1) and (C2) of Theorem 1. The rank of the discrete polymatroid is equal to five since the vector spaces $V_{1},V_{2},\ldots ,V_{5}$ are linearly independent. The rank of the vector space $V_{6}$ is equal to three, which is the length of the index code. We illustrate condition (C2) for receiver $R_{5}$. For receiver $R_{5}$, the matrices $AD_{5},AK_{5}$ and $A_{6}$ are as follows: *

$$AD_{5}=\left[\begin{array}{c} 0\\ 0\\ 1\\ 1\\ 1 \end{array}\right],AK_{5}=\left[\begin{array}{cc} 0 & 1\\ 1 & 0\\ 0 & 1\\ 0 & 0\\ 0 & 0 \end{array}\right],A_{6}=\left[\begin{array}{ccc} 1 & 0 & 0\\ 1 & 0 & 0\\ 0 & 1 & 0\\ 0 & 1 & 0\\ 0 & 0 & 1 \end{array}\right].$$

*Clearly $AD_{5}$ lies in the column span of the matrix $\left[AK_{5}\;A_{6}\right]$. Condition (C2) can be similarly verified for every receiver.*

**Example** **8.** *Consider the generalized index coding problem with the message vector $X=[x_{1},x_{2},\ldots ,x_{5}]$, $x_{i}\in F_{2}$. There are five receivers $R_{1}=\left(x_{1},\left\{x_{2}+x_{5}\right\}\right),R_{2}=\left(x_{2},\left\{x_{1}+x_{3}\right\}\right),R_{3}=\left(x_{3},\left\{x_{2}+x_{4}\right\}\right),R_{4}=\left(x_{4},\left\{x_{3}+x_{5}\right\}\right)$ and $R_{5}=\left(x_{5},\left\{x_{1}+x_{2}\right\}\right)$. The generalized index coding problem has an index code over $F_{2}$ of length six and dimension two. Note that the index code considered is a vector linear index code. For a receiver $R_{i},i\in \left\lceil 5\right\rfloor$ the demand matrix $D_{i}$ is equal to $\left[\epsilon_{2i-1,10}\;\;\epsilon_{2i,10}\right]$ and the knowledge matrix $K_{i}=\left[\epsilon_{2i+1,10}+\epsilon_{2i-3,10}\;\;\epsilon_{2i+2,10}+\epsilon_{2i-2,10}\right]$ where the operations on the indices are modulo ten with 0=10. The knowledge matrix $K_{1}$ and the demand matrix $D_{1}$ are as given below: *

$$K_{1}=\left[\begin{array}{cc} 0 & 0\\ 0 & 0\\ 1 & 0\\ 0 & 1\\ 0 & 0\\ 0 & 0\\ 0 & 0\\ 0 & 0\\ 1 & 0\\ 0 & 1 \end{array}\right],D_{1}=\left[\begin{array}{cc} 1 & 0\\ 0 & 1\\ 0 & 0\\ 0 & 0\\ 0 & 0\\ 0 & 0\\ 0 & 0\\ 0 & 0\\ 0 & 0\\ 0 & 0 \end{array}\right].$$

*The vector linear index code of dimension two is described by the matrix*

$$L=\left[\begin{array}{cccccc} 1 & 0 & 0 & 1 & 0 & 0\\ 0 & 1 & 1 & 1 & 0 & 0\\ 0 & 1 & 1 & 0 & 0 & 1\\ 1 & 1 & 0 & 1 & 1 & 1\\ 0 & 0 & 0 & 1 & 1 & 0\\ 0 & 0 & 1 & 1 & 0 & 1\\ 0 & 0 & 0 & 0 & 0 & 1\\ 0 & 0 & 0 & 0 & 1 & 1\\ 0 & 1 & 0 & 0 & 0 & 0\\ 1 & 1 & 0 & 0 & 0 & 0 \end{array}\right].$$

*For i∈⌈5⌋, let $A_{i}$ be the matrix $\left[\epsilon_{2i-1,10}\;\;\epsilon_{2i,10}\right]$ and let $A_{6}=L$. Let $V_{i}$ denote the column span of $A_{i},i\in \left\lceil 6\right\rfloor$. The discrete polymatroid $D(V_{1},V_{2},\ldots ,V_{6})$ satisfies the conditions of Theorem 1. For i∈⌈5⌋ the dimension of vector space $V_{i}$ is equal to two. The rank of the discrete polymatroid is equal to ten since the vector spaces $V_{1},V_{2},\ldots ,V_{5}$ are linearly independent. The dimension of the vector space $V_{6}$ is equal to six, which is the length of the vector linear index code. For receiver $R_{1}=(x_{1},\left\{x_{2}+x_{5}\right\}$ the matrices $AD_{1}$ and $AK_{1}$ are $D_{1}$ and $K_{1}$, respectively. Observe that $D_{1}$ lies in the span of the concatenated matrix $\left[K_{1}\;L\right]$. Hence, condition (C2) is satisfied for receiver $R_{1}$. Condition (C2) can be similarly verified for all other receivers.*

## 5. Generalized Index Coding from Discrete Polymatroids

Discrete polymatroids can be viewed as a generalization of matroids, as explained in Section 3. In Section 4, we established the connections between a generalized index coding problem having a vector linear solution and representable discrete polymatroids. In this section, starting from a discrete polymatroid, we construct generalized index coding problems. This was motivated by the previous works, which construct index coding problems from matroids [24] and discrete polymatroids [28]. We establish a connection between an optimal perfect linear solution for the constructed generalized index coding problems and representable discrete polymatroids in Theorem 2. This technique helps to generate a class of generalized index coding problems and also helps in identifying representations of discrete polymatroids from the solutions of generalized index coding problems.

Consider a discrete polymatroid D on the ground set ⌈r⌋ with rank function ρ and ρ(⌈r⌋)=k. The generalized index coding problem $I_{D}\left(Z,R\right)$ is given below.

(i) The set of source messages Z=X∪Y, where $X=\{x_{1},x_{2},\ldots ,x_{k}\}$ and
  $Y=\{y_{1}^{1},y_{1}^{2},\ldots ,y_{1}^{\rho (\{1\})},y_{2}^{1},y_{2}^{2},\ldots ,y_{2}^{\rho (\{2\})},\ldots ,y_{r}^{1},y_{r}^{2},\ldots ,y_{r}^{\rho (\{r\})}\}$.
(ii) The set of receivers R is a union of three sets of receivers $R_{1},R_{2}$ and $R_{3}$ defined below. Let $\zeta_{i}=\left\{y_{i}^{1},y_{i}^{2},\ldots ,y_{i}^{\rho (\{i\})}\right\}$.
  **Receivers in $R_{1}$: ** For a basis vector $b=\sum_{i\in \lceil r\rfloor }b_{i}\epsilon_{i,r}\in B\left(D\right)$, we define the set $S_{1}\left(b\right)=\{\left(x_{j},\underset{l\in {\left(b\right)}_{>0}}{\cup }\eta_{l}\right)\;\mathrm{for}\;\mathrm{all}\;j\in \left\lceil k\right\rfloor \;\mathrm{and}\;\mathrm{for}\;\mathrm{all}\;\eta_{l}\subseteq \zeta_{l}\;\mathrm{such}\;\mathrm{that}\;|\eta_{l}\left|=b_{l}\right\}$. $R_{1}=\underset{b\in B(D)}{\cup }S_{1}\left(b\right)$ is the union of all such receivers for every basis of the discrete polymatroid D.
  **Receivers in $R_{2}$: ** For a minimal excluded vector $c=\sum_{i\in \lceil r\rfloor }c_{i}\epsilon_{i,r}\in C\left(D\right),j\in {\left(c\right)}_{>0}$ and p∈⌈ρ({j})⌋, define the set $S_{2}\left(c,j,p\right)$ as follows:
  $$\begin{array}{c} S_{2}\left(c,j,p\right)=\{\left(y_{j}^{p},\underset{y\in \Gamma_{1}\cup \Gamma_{2}}{\sum y}\right):\Gamma_{1}=\underset{l\in {\left(c\right)}_{>0}\setminus \left\{j\right\}}{\cup }\eta_{l},\eta_{l}\subseteq \zeta_{l},|\eta_{l}|=c_{l},\Gamma_{2}\subseteq \zeta_{j}\setminus \left\{y_{j}^{p}\right\},|\Gamma_{2}\left|=c_{j}-1\right\}. \end{array}$$
  Define $R_{2}=\underset{c\in C(D)}{\cup }\;\;\underset{j\in {\left(c\right)}_{>0}}{\cup }\;\;\underset{p\in \lceil \rho (\{j\})\rfloor }{\cup }S_{2}\left(c,j,p\right).$
  **Receivers in $R_{3}$: ** Define $R_{3}=\{\left(y_{i}^{j},X\right):i\in \left\lceil r\right\rfloor ,j\in \left\lceil \rho \left(\left\{i\right\}\right)\right\rfloor$ }.

The presence of receivers in the set $R_{3}$ ensures that the minimum number of transmissions required by the above problem is $n\sum_{i\in \lceil r\rfloor }\rho \left(\left\{i\right\}\right)$. Note that the receivers in $R_{3}$ have the same *Has-set* and $\mu \left(I_{D}\left(X,R\right)\right)=\sum_{i\in \lceil r\rfloor }\rho \left(\left\{i\right\}\right)$. The construction is similar to the construction provided in [28]. The difference is in the set of receivers constructed from minimal excluded vectors of the discrete polymatroid D. The set of receivers in $R_{1}$ ensures that if a linear index coding solution exists then, from the messages corresponding to the elements in the basis of the discrete polymatroid and the transmitted messages, $x_{j},j\in \left\lceil k\right\rfloor$ is decodable. Receivers in $R_{2}$ have a function of messages as its side-information. Corresponding to every minimal excluded vector, a set of receivers is constructed, which captures the dependency existing in the minimal excluded vector. In this paper, receivers belonging to the set $R_{2}$ have a function of messages as its side information. The construction ensures that all the dependencies exiting in the minimal excluded vectors are captured by the receivers. The proof in [28] is modified for the case of these new set of receivers. We show that there exists a linear index coding solution, then we show that a representation can be constructed from the linear index code. The construction of receivers from a discrete polymatroid is made clear in the example below.

**Example** **9.** *Consider the discrete polymatroid D on the ground set ⌈3⌋ with the rank function ρ given by ρ{1}=ρ{2}=1,ρ{1,2}=ρ{3}=2 and ρ{1,3}=ρ{2,3}=ρ{1,2,3}=3. From the discrete polymatroid D we construct the generalized index coding problem $I_{D}\left(Z,R\right)$.*

*The set of messages possessed by the source is $Z=\left\{x_{1},x_{2},x_{3}\right\}\cup \left\{y_{1}^{1},y_{2}^{1},y_{3}^{1},y_{3}^{2}\right\}$. The set of receivers are constructed as in the theorem above. The set of basis vectors of the discrete polymatroid D is B(D)={(1,1,1),(1,0,2),(0,1,2)}. We have,*

$$\begin{array}{cc} S_{1}\left(\left(1,1,1\right)\right) & =\{\left(x_{i},\left\{y_{1}^{1},y_{2}^{1},y_{3}^{j}\right\}\right):i\in \left\lceil 3\right\rfloor ,j\in \left\lceil 2\right\rfloor \},\\ S_{1}\left(\left(1,0,2\right)\right) & =\{\left(x_{i},\left\{y_{1}^{1},y_{3}^{1},y_{3}^{2}\right\}\right):i\in \left\lceil 3\right\rfloor \},\\ S_{1}\left(\left(0,1,2\right)\right) & =\{\left(x_{i},\left\{y_{2}^{1},y_{3}^{1},y_{3}^{2}\right\}\right):i\in \left\lceil 3\right\rfloor \},\;\mathrm{and}\;\\ R_{1}=S_{1}((1, & 1,1))\cup S_{1}\left(\left(1,0,2\right)\right)\cup S_{1}\left(\left(0,1,2\right)\right). \end{array}$$

*There is only one excluded vector (1,1,2). We have,*

$$\begin{array}{cc} S_{2}\left(\left(1,1,2\right),1,1\right) & =\{\left(y_{1}^{1},\left\{y_{2}^{1}+y_{3}^{1}+y_{3}^{2}\right\}\right)\},\\ S_{2}\left(\left(1,1,2\right),2,1\right) & =\{\left(y_{2}^{1},\left\{y_{1}^{1}+y_{3}^{1}+y_{3}^{2}\right\}\right)\},\\ S_{2}\left(\left(1,1,2\right),3,1\right) & =\{\left(y_{3}^{1},\left\{y_{1}^{1}+y_{2}^{1}+y_{3}^{2}\right\}\right)\}, \end{array}$$

$$\begin{array}{cc} S_{2}\left(\left(1,1,2\right),3,2\right) & =\{\left(y_{3}^{2},\left\{y_{1}^{1}+y_{2}^{1}+y_{3}^{2}\right\}\right)\},\;\mathrm{and}\;\\ R_{2} & =\;\underset{j\in {\left(c\right)}_{>0}}{⋃}\;\underset{p\in \lceil \rho (\{j\})\rfloor }{⋃}\;S_{2}\left(\left(1,1,0\right),j,p\right). \end{array}$$

*Third set of receivers $R_{3}$ is a collection of four receivers $\left(y_{1}^{1},X\right),\left(y_{2}^{1},X\right),\left(y_{3}^{1},X\right)$ and $\left(y_{3}^{2},X\right)$ where $X=\{x_{1},x_{2},x_{3}\}$. From the set of receivers in $R_{3}$ it is clear that $\mu (I_{D}\left(Z.R\right))=4.$*

A connection between an optimal perfect linear solution for the generalized index coding problem $I_{D}\left(Z,R\right)$ and the representability of the discrete polymatroid D is established in Theorem 2 below.

**Theorem** **2.** *If an optimal perfect linear index coding solution of dimension n over $F_{2}$ exists for the generalized index coding problem $I_{D}\left(Z,R\right)$, then the discrete polymatroid nD is representable over $F_{2}$.*

**Proof.**  Let $t=k+\sum_{i=1}^{r}\rho \left(\left\{i\right\}\right)$ denote the number of messages in the index coding problem $I_{D}\left(Z,R\right)$. If an optimal perfect linear index coding solution of dimension *n* over $F_{2}$ exists for the index coding problem $I_{D}\left(Z,R\right)$, then from Theorem 1, there exists a discrete polymatroid $D^{\prime }$ representable over $F_{2}$ satisfying conditions (C1) and (C2). Discrete polymatroid $D^{\prime }$ has rank nt and is over the ground set ⌈t+1⌋. Let $V_{1},V_{2},\ldots ,V_{t+1}$ be the vector spaces over $F_{2}$, which form the representation of $D^{\prime }$. The vector spaces $V_{i},i\in \left\lceil t\right\rfloor$ can be expressed as the column span of matrices $A_{i}$ of order nt×n. The vector space $V_{t+1}$ can be written as the column span of $A_{t+1}$ of order $nt\times n\sum_{i=1}^{r}\rho \left(\left\{i\right\}\right)$. This is because the linear index code for the index coding problem is perfect. The matrix $B=[A_{1},A_{2},\ldots ,A_{t}]$ is invertible from (C1). We can assume it to be the identity without loss of generality. Otherwise, define $A_{i}^{\prime }=B^{-1}A_{i},i\in \left\lceil t+1\right\rfloor$ and vector spaces given by column spans of $A_{i}^{\prime }$ will form a representation of $D^{\prime }$. This is possible because pre multiplication by a full rank matrix does not change the discrete polymatroid.

The matrix $A_{t+1}$ is a $nt\times n\sum_{i=1}^{r}\rho \left(\left\{i\right\}\right)$ matrix and we can also assume the matrix to have a specific structure. This is because the presence of receivers belonging to $R_{3}$. Let $A_{t+1}={\left[C^{T}D^{T}\right]}^{T}$ where *C* is of order $nk\times n\sum_{i=1}^{r}\rho \left(\left\{i\right\}\right)$ and *D* is of order $\left(n\sum_{i=1}^{r}\rho \left(\left\{i\right\}\right)\right)\times \left(n\sum_{i=1}^{r}\rho \left(\left\{i\right\}\right)\right)$. The matrix $A=[A_{1}A_{2}\ldots A_{t+1}]$ is of the form $A=\left[\begin{array}{ccc} I_{nk} & 0 & C\\ 0 & I_{n(t-k)} & D \end{array}\right]$. The presence of receivers in $R_{3}$ ensures that the columns corresponding to the messages $y_{i}^{j},i\in \left\lceil r\right\rfloor ,j\in \left\lceil \rho \left(\left\{i\right\}\right)\right\rfloor$ lies in the linear span of the columns corresponding to messages *X* and the columns of the matrix $A_{t+1}$. We have

$$nt=\;\mathrm{rank}\;\left[\begin{array}{ccc} I_{nk} & 0 & C\\ 0 & I_{n(t-k)} & D \end{array}\right]=\;\mathrm{rank}\;\left[\begin{array}{cc} I_{nk} & C\\ 0 & D \end{array}\right]=nk+rank\left[D\right].$$

From this it follows that the matrix *D* has rank n(t−k) and hence it is a full rank matrix. We can assume *D* to be the identity because if not we can define $A_{t+1}^{\prime }=A_{t}D^{-1}$ and it still continues to be a valid representation. Let $C_{i},i\in \left\lceil r\right\rfloor$ denote the matrix obtained by taking only the ${\left(n\sum_{j=1}^{i-1}\rho \left(\left\{j\right\}\right)+1\right)}^{\mathrm{th}}$ to ${\left(n\sum_{j=1}^{i}\rho \left(i\right)\right)}^{\mathrm{th}}$ columns of *C*. Let $C_{i,j},j\in \left\lceil \rho \left(\left\{i\right\}\right)\right\rfloor$ denote the nk×n matrix obtained by taking the ${\left(\left(j-1\right)n+1\right)}^{\mathrm{th}}$ to ${\left(jn\right)}^{\mathrm{th}}$ columns of $C_{i}$. Let $V_{i}^{\prime }$ denote the column span of $C_{i}$ and $V_{i,j}^{\prime }$ denote the column span of $C_{i,j}$. We show that the vector subspaces $V_{i}^{\prime },i\in \left\lceil r\right\rfloor$ form a representation for the discrete polymatroid nD.

Consider a set S⊆⌈r⌋ with |S|=l. Let $b^{S}=\arg\max_{b\in D}\left|b\left(S\right)\right|$. Let $b_{i}^{S}$ denote the $i^{th}$ component of $b^{S}$. Choose $b_{i}^{S}$ independent vector subspaces from the set $V_{i}=\left\{V_{i,j}^{\prime }:j\in \left\lceil \rho \left(\left\{i\right\}\right)\right\rfloor \right\}$, denoted as $V_{i,o_{1}}^{\prime },V_{i,o_{2}}^{\prime },\ldots ,V_{i,o_{b_{i}^{S}}}^{\prime }$ for every i∈⌈r⌋. These vectors can be chosen because the set $V_{i}$ contains ρ({i}) independent vector subspaces. Note that $b_{i}^{S}<\rho \left(\left\{i\right\}\right)$, since b∈D. Let $\hat{V_{i}}=\sum_{j\in \lceil b_{i}^{S}\rfloor }V_{i,o_{j}}^{\prime }$ and $\hat{C_{i}}=\sum_{j\in \lceil b_{i}^{S}\rfloor }C_{i,o_{j}}$. The vector $\hat{C_{i}}$ is a column vector, which is the sum of $|b_{i}^{S}|$ column vectors of matrix $C_{i}$. The receivers in $S_{1}\left(b^{S}\right)$ demands the messages *X* and possess messages $b_{i}^{S}$ messages from the set of messages $y_{i}^{j},j\in \rho \left(\left\{i\right\}\right)$ for all i∈⌈r⌋. The demand matrix of these receivers is $I_{nk}$. From the fact that (C2) needs to be satisfied for the receivers belonging to $S_{1}\left(b^{S}\right)$, we have $dim\left(\sum_{i\in \lceil r\rfloor }\hat{V_{i}}\right)=\;\mathrm{rank}\;\left[I_{nk}\right]=nk=n\rho \left(D\right)$. This implies that $dim\left(\sum_{i\in S}\hat{V_{i}}\right)=n\left|b^{S}\left(S\right)\right|$. Since the vector space $\hat{V_{i}}$ is a subspace of $V_{i}^{\prime }$, we have $dim\left(\sum_{i\in S}V_{i}^{\prime }\right)\geq n\rho \left(S\right).$

The elements of the subset *S* can be divided into two categories depending upon the value of $b_{i}^{S}$. The first category corresponds to the elements in ground set for which $b_{s_{i}}^{S}<\rho \left(\left\{s_{i}\right\}\right)$ and the second category is the set of elements in ground set for which $b_{s_{i}}^{S}=\rho \left(\left\{s_{i}\right\}\right)$. Thus elements of *S* can be written as $S=\left\{s_{1},s_{2},\ldots ,s_{m}\right\}\cup \left\{s_{m+1},s_{m+2},\ldots ,s_{l}\right\}$, where $s_{1},s_{2}\ldots ,s_{m}$ are the elements for which $b_{s_{i}}^{S}<\rho \left(\left\{s_{i}\right\}\right)$ and the remaining elements satisfies $b_{s_{i}}^{S}=\rho \left(\left\{s_{i}\right\}\right)$. Consider the vector $u=\left(b_{s_{1}}^{S}+1\right)\epsilon_{s_{1},r}+\sum_{i\in S\setminus \{s_{1}\}}b_{i}^{S}\epsilon_{i,r}$. Since the vector does not belong to the discrete polymatroid it is an excluded vector. This implies that there exists a minimum excluded vector $u_{m}$ for which $u_{m}\leq u$. The $s_{1}^{\mathrm{th}}$ component of $u_{m}$ has to be $b_{s_{1}}^{S}+1$, otherwise $u_{m}<b^{S}$ and $u_{m}$ cannot be an excluded vector. The vector $u_{m}$ can be written as $\left(b_{s_{1}}^{S}+1\right)\epsilon_{s_{1},r}+\sum_{i\in S\setminus s_{1}}c_{i}^{S}\epsilon_{i,r},$ where $c_{i}^{S}\leq b_{i}^{S}$. From the receivers belonging to the set $S_{2}\left(u_{m},s_{1},p\right),$ where $p\in \left\lceil \rho \left(\left\{s_{1}\right\}\right)\right\rfloor \setminus \left\{o_{1},o_{2},\ldots ,o_{b_{s_{1}}^{S}}\right\}$, it follows that

$$C_{s_{1},p}=\left(\sum_{i\in {\left(u_{m}\right)}_{>0}\setminus \left\{s_{1}\right\}}\hat{C_{i}}\;\right)+\hat{C_{s_{1}}}.$$

This is true for every $p\in \left\lceil \rho \left(\left\{s_{1}\right\}\right)\right\rfloor \setminus \left\{o_{1},o_{2},\ldots ,o_{b_{s_{1}}^{S}}\right\}$. Note that the vector space $V_{s_{1},p}$ is the column span of matrix $C_{s_{1},p}$. It is true for any $|b_{s_{1}}^{S}|$ columns chosen in $\hat{C_{s_{1}}}$. It follows that the vector space $V_{s_{1},p}^{\prime }$ is a subspace of $\sum_{i\in {\left(u_{m}\right)}_{>}0}\hat{V_{i}}$ for all $p\in \rho (\left\{s_{1}\right\})$. From this, we obtain that $\sum_{p\in \lceil \rho \left(\left\{s_{1}\right\}\right)\rfloor }V_{s_{1},p}^{\prime }\subseteq \sum_{i\in {\left(u_{m}\right)}_{>}0}\hat{V_{i}}\subseteq \sum_{i\in S}\hat{V_{i}}$. By a similar reasoning, $V_{s_{j}}^{\prime }\subseteq \sum_{i\in S}\hat{V_{i}},\forall j\in \left\lceil m\right\rfloor$. Since $b_{s_{j}}^{S}=\rho \left(\left\{s_{j}\right\}\right)$, for j∈{m+1,m+2,…,l}, we have $V_{s_{j}}^{\prime }=\hat{V_{s_{j}}}$ for j∈{m+1,m+2,…,l}. From the above facts we have $\sum_{i\in S}V_{i}^{\prime }\subseteq \sum_{i\in S}\hat{V_{i}}$. Hence, $dim\left(\sum_{i\in S}V_{i}^{\prime }\right)\leq dim\left(\sum_{i\in S}\hat{V_{i}}\right)=n\rho \left(S\right)$. Thus, we have established that $dim\left(\sum_{i\in S}V_{i}^{\prime }\right)=n\rho \left(S\right)$ for an arbitrary subset S⊆⌈r⌋. □

In Theorem 2, a generalized index coding problem is constructed from a discrete polymatroid and then it is shown that the discrete polymatroid is representable over the field $F_{2}$ if an optimal perfect linear index coding solution exists for the constructed generalized index coding problem. The proof of Theorem 2 follows closely the proof of a similar theorem in [28]. This is because in the constructed generalized index coding problem only the receivers constructed from minimal excluded vectors differ. We illustrate this theorem in Example 10. The converse of this result is however not true. In Example 11, from a binary representable discrete polymatroid we construct a generalized index coding problem for which there is no optimal perfect linear index coding solution.

**Example** **10.** *Consider the discrete polymatroid described in Example 9. The set of receivers constructed from the discrete polymatroid is provided in Example 9. Consider the perfect index code in which the source transmits $y_{1}^{1}+x_{1},y_{2}^{1}+x_{2},y_{3}^{1}+x_{3}$ and $y_{3}^{2}+x_{1}+x_{2}+x_{3}$. From Theorem 1 there exists a representable discrete polymatroid on the ground set ⌈8⌋. Each element i∈⌈8⌋ is represented by the column space of the matrix $A_{i}$. For $i\in \left\lceil 7\right\rfloor ,A_{i}=\epsilon_{i,7}$ and*

$$A_{8}=\left[\begin{array}{cccc} 1 & 0 & 0 & 1\\ 0 & 1 & 0 & 1\\ 0 & 0 & 1 & 1\\ 1 & 0 & 0 & 0\\ 0 & 1 & 0 & 0\\ 0 & 0 & 1 & 0\\ 0 & 0 & 0 & 0 \end{array}\right].$$

*It was shown in the proof of Theorem 2 that the matrix $A_{8}$ has a specific structure of the form ${\left[C^{T}D^{T}\right]}^{T}$ with the matrix D taking the form of an identity matrix. It was also shown that the matrix C forms a valid representation of the discrete polymatroid D. From the structure of $A_{8}$ we have*

$$C=\begin{array}{c} \left[\begin{array}{cccc} 1 & 0 & 0 & 1\\ 0 & 1 & 0 & 1\\ 0 & 0 & 1 & 1 \end{array}\right]\\ \underset{C_{1}}{︸}\underset{C_{2}}{︸}\underset{C_{3}}{︸} \end{array}.$$

*It can be verified that the matrix C is a representing matrix for the discrete polymatroid D.*

**Example** **11.** *Consider the discrete polymatroid D on the ground set ⌈3⌋ with the rank function ρ given by ρ{1}=ρ{2}=ρ{2,3}=2,ρ{3}=1 and ρ{1,2}=ρ{1,3}=ρ{1,2,3}=3. The generalized index coding problem $I_{D}\left(Z,R\right)$ constructed from the discrete polymatroid is as follows. The set of messages $Z=\left\{x_{1},x_{2},x_{3}\right\}\cup \left\{y_{1}^{1},y_{1}^{2},y_{2}^{1},y_{2}^{2},y_{3}^{1}\right\}$.*

*The set of basis vectors for the problem is B(D)={(1,1,1),(1,2,0),(2,0,1),(2,1,0)}. We have,*

$$\begin{array}{cc} S_{1}\left(\left(1,1,1\right)\right) & =\{\left(x_{i},\left\{y_{1}^{j},y_{2}^{k},y_{3}^{1}\right\}\right):i\in \left\lceil 3\right\rfloor ,j,k\in \left\lceil 2\right\rfloor \},\\ S_{1}\left(\left(1,2,0\right)\right) & =\{\left(x_{i},\left\{y_{1}^{j},y_{2}^{1},y_{2}^{2}\right\}\right):i\in \left\lceil 3\right\rfloor ,j\in \left\lceil 2\right\rfloor \},\\ S_{1}\left(\left(2,0,1\right)\right) & =\{\left(x_{i},\left\{y_{1}^{1},y_{1}^{2},y_{3}^{1}\right\}\right):i\in \left\lceil 3\right\rfloor \},\\ S_{1}\left(\left(2,1,0\right)\right) & =\{\left(x_{i},\left\{y_{1}^{1},y_{1}^{2},y_{2}^{j}\right\}\right):i\in \left\lceil 3\right\rfloor ,j\in \left\lceil 2\right\rfloor \}\;\mathrm{and}\;\\ R_{1}=S_{1}((1, & 1,1))\cup S_{1}\left(\left(1,2,0\right)\right)\cup S_{1}\left(\left(2,0,1\right)\right)\cup S_{1}\left(\left(2,1,0\right)\right). \end{array}$$

*The set of minimal excluded vectors of D are $c_{1}=\left(0,2,1\right),c_{2}=\left(2,1,1\right)$ and $c_{3}=\left(2,2,0\right)$. We have,*

$$\begin{array}{cc} S_{2}\left(c_{1},2,1\right) & =\{\left(y_{2}^{1},\left\{y_{2}^{2}+y_{3}^{1}\right\}\right)\},\\ S_{2}\left(c_{1},2,2\right) & =\{\left(y_{2}^{2},\left\{y_{2}^{1}+y_{3}^{1}\right\}\right)\},\\ S_{2}\left(c_{1},3,1\right) & =\{\left(y_{3}^{1},\left\{y_{2}^{1}+y_{2}^{2}\right\}\right)\},\\ S_{2}\left(c_{2},1,1\right) & =\{\left(y_{1}^{1},\left\{y_{1}^{2}+y_{2}^{i}+y_{3}^{1}\right\}\right):i\in \left\lceil 2\right\rfloor \},\\ S_{2}\left(c_{2},1,2\right) & =\{\left(y_{1}^{2},\left\{y_{1}^{1}+y_{2}^{i}+y_{3}^{1}\right\}\right):i\in \left\lceil 2\right\rfloor \},\\ S_{2}\left(c_{2},2,1\right) & =\{\left(y_{2}^{1},\left\{y_{1}^{1}+y_{1}^{2}+y_{3}^{1}\right\}\right)\},\\ S_{2}\left(c_{2},2,2\right) & =\{\left(y_{2}^{2},\left\{y_{1}^{1}+y_{1}^{2}+y_{3}^{1}\right\}\right)\},\\ S_{2}\left(c_{2},3,1\right) & =\{\left(y_{3}^{1},\left\{y_{1}^{1}+y_{1}^{2}+y_{2}^{i}\right\}\right):i\in \left\lceil 2\right\rfloor \},\\ S_{2}\left(c_{3},1,1\right) & =\{\left(y_{1}^{1},\left\{y_{1}^{2}+y_{2}^{1}+y_{2}^{2}\right\}\right)\},\\ S_{2}\left(c_{3},1,2\right) & =\{\left(y_{1}^{2},\left\{y_{1}^{1}+y_{2}^{1}+y_{2}^{2}\right\}\right)\},\\ S_{2}\left(c_{3},2,1\right) & =\{\left(y_{2}^{1},\left\{y_{1}^{1}+y_{1}^{2}+y_{2}^{2}\right\}\right)\},\\ S_{2}\left(c_{3},2,2\right) & =\{\left(y_{2}^{2},\left\{y_{1}^{1}+y_{1}^{2}+y_{2}^{1}\right\}\right)\}\;\mathrm{and}\;\\ R_{2}=\underset{c\in \{c_{1},c_{2},c_{3}\}}{⋃} & \;\underset{j\in {\left(c\right)}_{>0}}{⋃}\;\underset{p\in \lceil \rho (\{j\})\rfloor }{⋃}\;S_{2}\left(c,j,p\right). \end{array}$$

*Third set of receivers $R_{3}$ is a collection of five receivers $\left(y_{1}^{1},X\right),\left(y_{1}^{2},X\right),\left(y_{2}^{1},X\right),\left(y_{2}^{2},X\right),\left(y_{3}^{1},X\right)$ where $X=\{x_{1},x_{2},x_{3}\}$.*

*The discrete polymatroid D has a binary representation given by the representing matrix*

$$A=\begin{array}{c} \left[\begin{array}{ccccc} 1 & 0 & 0 & 1 & 0\;\\ 0 & 1 & 0 & 1 & 0\;\\ 0 & 0 & 1 & 1 & 1\; \end{array}\right]\\ \;\underset{A_{1}}{︸}\;\underset{A_{2}}{︸}\;\underset{A_{3}}{︸} \end{array}.$$

*Though the discrete polymatroid has a binary representation, the generalized index coding problem $I_{D}\left(Z,R\right)$ constructed from it does not have a perfect binary solution. Suppose there exists an optimal scalar perfect linear solution over $F_{2}$. From Theorem 2, an optimal scalar perfect linear solution exists only if D is representable over $F_{2}$. Every optimal scalar perfect linear solution for $I_{D}\left(Z,R\right)$ can be written as $f\left(Z\right)=\left[y_{1}^{1}\;y_{1}^{2}\;y_{2}^{1}\;y_{2}^{2}\;y_{3}^{1}\right]A+\left[x_{1}\;x_{2}\;x_{3}\right]G$ where A is a 5×5 matrix over $F_{2}$ and G is a 3×5 matrix over $F_{2}$. The matrix A needs to be full rank to ensure that the receivers belonging to $R_{3}$ are satisfied, which allows us to assume A to be the identity matrix. It can be shown by checking all possible solutions that there does not exist a 3×5 matrix G over $F_{2}$ that solves the generalized index coding problem. We provide an alternate proof here. Let $G_{i}$ denote the $i^{th}$ column of G. The first three columns of the matrix G can be assumed to be the columns of the 3×3 identity matrix. The column $G_{5}$ has to be ${\left[1\;1\;1\right]}^{T}$. The column $G_{5}$ cannot be ${\left[1\;0\;0\right]}^{T},{\left[0\;1\;0\right]}^{T}$ and ${\left[1\;1\;0\right]}^{T}$, since $dim(V_{1}+V_{3})=3$. If $G_{5}={\left[0\;1\;1\right]}^{T}$, the receivers $\left(x_{i},\left\{y_{1}^{2},y_{2}^{1},y_{3}^{1}\right\}\right),i\in \left\lceil 3\right\rfloor$ fail to decode the demands. Similarly if $G_{5}={\left[0\;1\;1\right]}^{T}$, the receivers $\left(x_{i},\left\{y_{1}^{1},y_{2}^{1},y_{3}^{1}\right\}\right),i\in \left\lceil 3\right\rfloor$ fail and if $G_{5}={\left[1\;0\;1\right]}^{T}$, the receivers $\left(x_{i},\left\{y_{1}^{1},y_{2}^{1},y_{3}^{1}\right\}\right),i\in \left\lceil 3\right\rfloor$ fail to decode the demands. From the restrictions $dim(V_{2}+V_{3})=2$ and $dim(V_{2})=2$, the column vector $G_{4}$ has only two possibilities ${\left[1\;1\;0\right]}^{T}$ and ${\left[1\;1\;1\right]}^{T}$. If $G_{4}$ is equal to ${\left[1\;1\;0\right]}^{T}$, then receivers $\left(x_{i},\left\{y_{1}^{1},y_{1}^{2},y_{2}^{2}\right\}\right)$ fail and if $G_{4}={\left[1\;1\;1\right]}^{T}$, then receivers $\left(x_{i},\left\{y_{1}^{1}.y_{2}^{2},y_{3}^{1}\right\}\right)$ fail to decode the demands. This shows that there does not exist an optimal perfect scalar linear solution over $F_{2}$ for $I_{D}\left(Z,R\right)$.*

## 6. Matroids and Generalized Index Coding Problems

In this section, we construct a generalized index coding problem from a matroid. The construction explained in Section 5 is more general than this since discrete polymatroids can be viewed as a generalization of matroids. The advantage of this construction is the existence of an if and only if the relationship between the constructed index coding problem and the representability of the matroids as shown in Theorem 3. The construction enables us to start with any representable matroid and convert it to a generalized index coding problem with optimal perfect solutions. This paves the way for the construction of a large class of generalized index coding problems with optimal perfect solutions. The index code constructed from the matroid is similar to the construction provided in [24]. Receivers belonging to the set $R_{2}$, which are constructed from the circuits of matroid are different, as explained below.

**Definition** **7.** *Given a matroid M(Y,r) of rank k over the ground set $Y=\{y_{1},\cdots ,y_{m}\}$, we define a corresponding index coding with coded side information problem $I_{M}\left(Z,R\right)$ as follows:*

*1.*  *Z=Y∪X, where $X=\{x_{1},\cdots ,x_{k}\}$,*
*2.*  *$R=R_{1}\cup R_{2}\cup R_{3}$ where*
  *(a)*  $R_{1}=\left\{\left(x_{i},B\right);B\in B\left(M\right),i=1,\cdots ,k\right\}$
  *(b)*  $R_{2}=\left\{\left(y,\sum_{y_{j}\in C\setminus \left\{y\right\}}y_{j}\right);C\in C\left(M\right),y\in C\right\}$
  *(c)*  $R_{3}=\left\{\left(y_{i},X\right);i=1,\cdots ,m\right\}$

There is a one to one correspondence between a matroid M and the discrete polymatroid D(M). From the discrete polymatroid D(M), we can construct a generalized index coding problem $I_{D(M)}\left(Z,R\right)$. The generalized index coding problem $I_{D(M)}\left(Z,R\right)$ reduces to the generalized index coding problem $I_{M}\left(Z,R\right)$. The rank of every element in the ground set is one and hence the set *Y* remains the same in both $I_{D(M)}\left(Z,R\right)$ and $I_{M}\left(Z,R\right)$. It can also be seen that the receivers $R_{1},R_{2}$ and $R_{3}$ are same for $I_{D(M)}\left(Z,R\right)$ and $I_{M}\left(Z,R\right)$.

**Remark** **1.** *Every receiver in an index coding problem introduces a dependency between demanded messages, messages available as side information and transmitted messages. Matroids and discrete polymatroids captures these dependencies existing between its elements. Each message and the transmitted index code are viewed as elements in the ground set and the dependency between them is captured. However, for the set of dependent elements to characterize a matroid, it needs to satisfy certain axioms referred to as circuit axioms provided in the paper. The generalized index coding problem constructed in Definition 7 captures all the dependencies.*

**Theorem** **3.** *Consider a matroid M(Y,r) on the ground set $Y=\{y_{1},\cdots ,y_{m}\}$, and $I_{M}\left(Z,R\right)$ be the corresponding generalized index coding problem constructed from it. Then, the matroid M has a linear representation over $F_{2}$ if and only if there exists an optimal perfect scalar linear index code for $I_{M}\left(Z,R\right)$ over $F_{2}$.*

**Proof.**  From Theorem 2 and from the fact that there is a one to one correspondence between discrete polymatroid D(M) and matroid M it follows that the matroid M has a linear representation over $F_{2}$ if there exists an optimal perfect scalar linear index code for $I_{M}\left(Z,R\right)$. We prove the only if part here. We first assume that the matroid M is representable and show the existence of an optimal perfect scalar linear index code for the index coding problem $I_{M}\left(Z,R\right)$.

Let *M* be a matrix representing the matroid M. Since the matroid M is of rank *k*, the matrix *M* is a k×m matrix. Let $\xi =\left(x_{1},\ldots ,x_{k}\right)\in F_{2}^{k}$, and $\chi =\left(y_{1},\ldots ,y_{m},x_{1},\cdots ,x_{k}\right)\in F_{2}^{\left(m+k\right)}.$

Consider the following linear map $f\left(\chi \right)=(f_{1}\left(\chi \right),\cdots ,f_{m}\left(\chi \right))$ where

$$f_{i}\left(\chi \right)=y_{i}+\xi M_{i}\in F_{2},i=1,\cdots ,m.$$

Note that *f* is a map from $F_{2}^{m+k}$ to $F_{2}^{m}$. We show that *f* is an optimal perfect scalar linear index code for $I_{M}\left(Z,R\right)$. To show this we show that all the receivers are able to satisfy their demands using their *Has-sets* and the transmitted messages.

- Receivers in $R_{1}$: Consider a basis $B=\left\{y_{i_{1}},\cdots ,y_{i_{k}}\right\}\in B\left(M\right)$, and let $\rho_{i}=\left(x_{i},B\right)\in R_{1}$, i=1,⋯,k. We have $f_{i_{j}}\left(\chi \right)=y_{i_{j}}+\xi M_{i_{j}},j=1,2,\ldots ,k$. Combining these equations we obtain
  $$\left[f_{i_{1}}\left(\chi \right)\;f_{i_{2}}\left(\chi \right)\ldots \;f_{i_{k}}\left(\chi \right)\right]=\left[y_{i_{1}}\;y_{i_{2}}\ldots \;y_{i_{k}}\right]+\xi \left[M_{i_{1}}M_{i_{2}}\ldots M_{i_{k}}\right].$$
  Since $\left\{y_{i_{1}},\cdots ,y_{i_{k}}\right\}\in B\left(M\right)$ the matrix formed by concatenation of $M_{i_{1}},M_{i_{2}},\ldots ,M_{i_{k}}$ is invertible. Let $B=[M_{i_{1}}\;M_{i_{2}}\ldots \;M_{i_{k}}]$. The receivers can obtain ξ using the relation that
  $$\xi =\left[f_{i_{1}}\left(\chi \right)-y_{i_{1}}\;f_{i_{2}}\left(\chi \right)-y_{i_{2}}\ldots f_{i_{k}}\left(\chi \right)-y_{i_{k}}\right]B^{-1}.$$
- Receivers in $R_{2}$: Let $C=\left\{y_{i_{1}},\cdots ,y_{i_{c}}\right\}\in C\left(M\right)$ and $\rho =\left(y_{i_{1}},\sum_{y_{j}\in C\setminus \left\{y_{i_{1}}\right\}}y_{j}\right)\in R_{2}$. Let $C^{\prime }=C\setminus y_{i_{1}}$. We have $f_{i_{j}}\left(\chi \right)=y_{i_{j}}+\xi M_{i_{j}},j=1,2,\ldots ,c$. From this we can establish the relation
  $$f_{i_{2}}\left(\chi \right)+\ldots +f_{i_{c}}\left(\chi \right)=y_{i_{2}}+\ldots +y_{i_{c}}+\xi \left(M_{i_{2}}+\ldots +M_{i_{c}}\right).$$
  Since the matroid is representable over a binary field, we have $M_{i_{1}}=M_{i_{2}}+M_{i_{3}}+\ldots +M_{i_{c}}$ and the receiver can decode its demanded message $y_{i_{1}}$ using the relation
  $$y_{i_{1}}=\left(f_{i_{1}}\left(\chi \right)+f_{i_{2}}\left(\chi \right)+\ldots +f_{i_{c}}\left(\chi \right)\right)+\left(y_{i_{2}}+\ldots +y_{i_{c}}\right).$$
  In a similar way, all receivers belonging to $R_{2}$ can decode their demanded messages.
- Receivers in $R_{3}$: For all $\rho =\left(y_{i},X\right)\in R_{3}$, receivers can obtain its demanded message using the relation $y_{i}=f_{i}\left(\chi \right)-\xi M_{i}$.

The index code is clearly linear and also $\mu (I_{M}\left(Z,R\right))=m$. Hence, the code defined by the map *f* is an optimal perfect linear index code. This completes the proof. □

Theorem 3 shows the existence of a relationship between binary representability of matroids and solutions to certain generalized index coding problems. Theorem 3 is illustrated in Example 12. We use the theorem to show that not every generalized index coding problem has a perfect binary solution in Example 13.

**Example** **12.** *The uniform matroid $U_{2,3}$ is defined on a ground set $Y=\{y_{1},y_{2},y_{3}\}$ of three elements, such that ∀I⊆Y and |I|≤2,r(I)=|I|, and r(Y)=2. Consider the binary linear representation of $U_{2,3}$ given by $M=\left[\begin{array}{ccc} 1 & 0 & 1\\ 0 & 1 & 1 \end{array}\right].$ The index coding with the coded side information problem corresponding to this matroid has the source messages set $\chi =\{y_{1},y_{2},y_{3},x_{1},x_{2}\}$, where each message belongs to the finite field $F_{2}$. There are three sets of receivers and they are given below.*

- *Receivers in $R_{1}$: $\left\{x_{1},\left\{y_{1},y_{2}\right\}\right\},\{x_{2},$$\left\{y_{1},y_{2}\}\right\}$, $\left\{x_{1},\left\{y_{1},y_{3}\right\}\right\}$, $\left\{x_{2},\left\{y_{1},y_{3}\right\}\right\}$, $\left\{x_{1},\left\{y_{2},y_{3}\right\}\right\}$, $\left\{x_{2},\left\{y_{2},y_{3}\right\}\right\}$.*
- *Receivers in $R_{2}$: $\left\{y_{1},\left\{y_{2}+y_{3}\right\}\right\},\left\{y_{1},\left\{y_{2}+y_{3}\right\}\right\}$,$\left\{y_{1},\left\{y_{2}+y_{3}\right\}\right\}$*
- *Receivers in $R_{3}$: $\left\{y_{1},\left\{x_{1},x_{2}\right\}\right\}$, $\left\{y_{2},\left\{x_{1},x_{2}\right\}\right\}$,$\left\{y_{3},\left\{x_{1},x_{2}\right\}\right\}$*

*The optimal perfect linear index coding solution for the index coding problem is given by the map $f:F_{2}^{5}\to F_{2}^{3}$ given by*

$$f\left(\chi \right)=\left[y_{1}\;y_{2}\;y_{3}\right]+\left[x_{1}\;x_{2}\right]M.$$

*The index code is as follows: $c_{1}=y_{1}+x_{1}$, $c_{2}=y_{2}+x_{2}$, $c_{3}=y_{3}+x_{1}+x_{2}$*

*A receiver $\left\{x_{i},\left\{y_{j},y_{k}\right\}\right\}\in R_{1}$ can decode its demand from the transmissions $c_{j}$ and $c_{k}$. Note that $c_{1}+c_{2}+c_{3}=y_{1}+y_{2}+y_{3}$. Receivers belonging to the set $R_{2}$ can decode its demands by adding the information available in its Has-set to $c_{1}+c_{2}+c_{3}$. The receivers $\left\{y_{1},\left\{x_{1},x_{2}\right\}\right\}$, $\left\{y_{2},\left\{x_{1},x_{2}\right\}\right\}$ and $\left\{y_{3},\left\{x_{1},x_{2}\right\}\right\}$ belonging to $R_{3}$ can decode its demands from the transmissions $c_{1},c_{2}$ and $c_{3}$, respectively. Thus, all the receivers in the index coding problem constructed from the uniform matroid $U_{2,3}$, are able to decode its required messages. This is made more explicit in [40].*

**Example** **13.** *Consider the index coding problem with coded side information I(Z,R) :*

*The set of messages $Z=\{y_{1},y_{2},y_{3},y_{4},x_{1},x_{2}\}$. The set of receivers are given below.*

- *Receivers in $R_{1}$: $\left\{\left(x_{i},\left\{y_{1},y_{2}\right\}\right),i\in \left\lceil 2\right\rfloor \right\},\left\{\left(x_{i},\left\{y_{1},y_{3}\right\}\right),i\in \left\lceil 2\right\rfloor \right\}$, $\left\{\left(x_{i},\left\{y_{1},y_{4}\right\}\right),i\in \left\lceil 2\right\rfloor \right\}$, $\left\{\left(x_{i},\left\{y_{2},y_{3}\right\}\right),i\in \left\lceil 2\right\rfloor \right\}$, $\left\{\left(x_{i},\left\{y_{2},y_{4}\right\}\right),i\in \left\lceil 2\right\rfloor \right\}$, $\left\{\left(x_{i},\left\{y_{3},y_{4}\right\}\right),i\in \left\lceil 2\right\rfloor \right\}$.*
- *Receivers in $R_{2}$: $\left\{y_{1},\left\{y_{2}+y_{3}\right\}\right\},\left\{y_{2},\left\{y_{1}+y_{3}\right\}\right\}$,$\left\{y_{3},\left\{y_{1}+y_{3}\right\}\right\}$,$\left\{y_{1},\left\{y_{2}+y_{4}\right\}\right\}$,$\left\{y_{2},\left\{y_{1}+y_{4}\right\}\right\}$, $\left\{y_{4},\left\{y_{1}+y_{2}\right\}\right\}$,$\left\{y_{1},\left\{y_{3}+y_{4}\right\}\right\}$,$\left\{y_{3},\left\{y_{1}+y_{4}\right\}\right\}$,$\left\{y_{4},\left\{y_{1}+y_{3}\right\}\right\}$,$\left\{y_{2},\left\{y_{3}+y_{4}\right\}\right\}$,$\left\{y_{3},\left\{y_{2}+y_{4}\right\}\right\}$ and $\left\{y_{4},\left\{y_{2}+y_{3}\right\}\right\}$*
- *Receivers in $R_{3}$: $\left\{\left(y_{i},\left\{x_{1},x_{2},x_{3},x_{4}\right),i\in \left\lceil 4\right\rfloor \right\}\right\}$.*

*The above index coding problem is constructed from the uniform matroid $U_{2,4}$. The uniform matroid is defined on a ground set $Y=\{y_{1},y_{2},y_{3},y_{4}\}$ such that ∀I⊆Y and |I|≤2,r(I)=|I|, and r(Y)=2. The matroid $U_{2,4}$ does not have a binary representation. The matroid has a representation over ternary field GF(3): *

$$V_{1}=\left[\begin{array}{c} 1\\ 0 \end{array}\right],V_{2}=\left[\begin{array}{c} 0\\ 1 \end{array}\right],V_{3}=\left[\begin{array}{c} 1\\ 1 \end{array}\right],V_{4}=\left[\begin{array}{c} 1\\ 2 \end{array}\right].$$

*It can be verified that the above generalized index coding problem does not have an optimal perfect scalar binary linear solution as implied by Theorem 3. Since the matroid does not have a linear representation over the binary field, the generalized index coding problem constructed from it does not have an optimal scalar perfect linear solution.*

## 7. Conclusions

In this work, we established a few connections between generalized index coding and discrete polymatroids. It is shown that the existence of a linear solution for a generalized index coding problem is connected to the existence of a representable discrete polymatroid satisfying certain conditions determined by the generalized index coding problem. From a discrete polymatroid, a corresponding generalized index coding problem is constructed and it is shown that a representation to the discrete polymatroid exists if an optimal perfect vector linear solution exists for the generalized index coding problem. An example is provided in the paper to illustrate that the converse of the above result is not true. When a similar generalized index coding problem is constructed from matroids, we show that a binary representation to the matroid exists if and only if the constructed index coding problem has an optimal binary scalar linear solution. The connection is helpful in determining whether the index coding problem has an optimal perfect binary scalar linear solution.

The results of this paper could be extended in the following directions. The construction explained in Section 5 is general and can be applied to any discrete polymatroid. A generalized index coding problem can be constructed from a non representable discrete polymatroid and further connections could be explored. Also for the constructed index coding problem, certain receivers (belonging to the set $R_{2}$) possess the sum of certain elements as its *Has-set*. The elements of the *Has-set* can be made into any other linear combinations and further study could be done. A possible direction is to construct a discrete polymatroid for any generalized index coding problems. There may exist generalized index coding problems having non-linear solutions for which a connection to a non-representable discrete polymatroid could be established. This has not yet been explored and requires further investigation. Similar extensions can be considered to Theorem 3. Connections between the matroids representable over a non binary field and the generalized index coding problems constructed from those matroids is also another open problem. The connections between error correcting index codes for generalized index coding problems and discrete polymatroids could also be explored.
